# Functionalized gold nanoflowers on carbon screen-printed electrodes: an electrochemical platform for biosensing hemagglutinin protein of influenza A H1N1 virus

**DOI:** 10.3762/bjnano.16.42

**Published:** 2025-04-16

**Authors:** Carlos Enrique Torres-Méndez, Sharmilee Nandi, Klara Martinovic, Patrizia Kühne, Yifan Liu, Sam Taylor, Maria Lysandrou, Maria Ines Berrojo Romeyro Mascarenhas, Viktoria Langwallner, Javier Enrique Sebastián Alonso, Ivana Jovanovic, Maike Lüftner, Georgia-Vasiliki Gkountana, David Bern, Abdul-Raouf Atif, Ehsan Manouchehri Doulabi, Gemma Mestres, Masood Kamali-Moghaddam

**Affiliations:** 1 Division of Biomedical Engineering, Department of Materials Science and Engineering, Uppsala University, Uppsala, Sweden; 2 Department of Immunology, Genetics and Pathology, Science for Life Laboratory, Uppsala University, Uppsala, Sweden

**Keywords:** charge transfer, cyclic voltammetry, differential pulse voltammetry, electrochemical impedance spectroscopy, electrodeposition

## Abstract

An electrochemical biosensor based on modified carbon screen-printed electrodes was developed for the detection of hemagglutinin of influenza A H1N1 virus (H1). Gold nanoflowers were electrodeposited on the electrode to increase conductivity and surface area. The electrochemical signal was amplified by functionalization of the gold nanoflowers with 4-aminothiophenol, which resulted in a 100-fold decrease of the charge transfer resistance due to a tunneling effect. Subsequently, monoclonal antibodies against H1 were immobilized on the surface via covalent amide bond formation, followed by blocking with bovine serum albumin to minimize nonspecific hydrophobic binding. The electrodes were characterized by cyclic voltammetry and electrochemical impedance spectroscopy experiments in the presence of [Fe(CN)_6_]^3−/4−^. Differential pulse voltammetry was used to measure the change in current across the electrode as a function of H1 concentration. This was performed on a series of samples of artificial saliva containing H1 protein in a clinically relevant concentration range. In these experiments, the biosensor showed a limit of detection of 19 pg/mL. Finally, the biosensor platform was coupled to an automated microfluidics system, and no significant decrease of the electrochemical signal was observed.

## Introduction

Viral infections pose a threat to medical and public health systems, and the economic expenditures due to viral infections have increased steadily for health care systems in past years [1]. Influenza is an acute respiratory disease in mammals and domestic poultry, which emerges from zoonotic reservoirs in aquatic birds and bats. Influenza viruses are capable of evolving at a fast rate; they have a segmented single-stranded negative-sense RNA genome that is devoid of proofreading systems, resulting in a constant accumulation of mutations in their genome [2]. Influenza viruses belong to the *Orthomyxoviridae* family and are categorized into four groups, namely, influenza A, B, C, and D viruses. The antigenic features of the hemagglutinin (HA) and neuraminidase (NA) glycoproteins on the surface of influenza A viruses are used to further classify the virus into subtypes. Influenza A comprises 18 HA subtypes and 11 NA subtypes, of which only the H1, H2, H3, N1 and N2 strains have been associated with widespread human epidemics [3]. H1 protein initiates infection by binding to the cell surface and inducing membrane fusion. This protein is considered as a prime determinant of the pathogenicity and is the most abundant influenza surface glycoprotein [4]. These features make H1 protein a great target for biosensing.

Traditionally, infections caused by influenza A H1N1 are diagnosed through viral culture, immunofluorescence assay, and enzyme-linked immunosorbent assay (ELISA) [5]. These techniques suffer from two key drawbacks. They require lengthy protocols that take a few days to complete, and they fail to detect the virus at early stages of infection because of the low concentration of viral particles. However, detection of viral infections at early stages of infection is essential to prevent the dissemination of pathogens and the emergence of future pandemics.

Recently, molecular methods capable of detecting viral pathogens have gained more attention because of their inherent high sensitivity and specificity compared to conventional methods. Among these methods, nucleic acid amplification assays such as reverse transcriptase polymerase chain reaction and loop‐mediated isothermal amplification assays have shown great sensitivity for the detection of influenza A virus. These techniques target the genetic material of the virus, and meticulous protocols are required to perform the extraction from the samples [5]. Moreover, they require highly specialized infrastructure built in place as well as trained professionals, making detection methods based on nucleic acid detection and amplification less accessible [6].

Rapid, sensitive, reliable, and easily available diagnostic methods for influenza A H1N1 virus are needed to detect infected patients at an early stage to improve treatment options, recovery time, and economic cost. Biosensors are widely used to detect and quantify different analytes. They incorporate a biorecognition element for detection of the analyte of interest in a sample and a physicochemical transducer to generate measurable signals that reflect the concentration of the analyte [7]. Among different types of biosensors, electrochemical biosensors are particularly advantageous, since they can be built from low-cost components, designed to be compact and portable, while preserving high resolution, accuracy, and sensitivity [8].

In the last few years, various biosensors for the detection of influenza A H1N1 virus have been developed. Detection of influenza A H1N1 virus can be achieved by targeting one or more relevant biomolecules of the virus. The majority of studies have targeted H1 protein [9–20], while others have focused on N1 protein [8,21], both H1 and N1 proteins [22], nucleoprotein [23–25], both H1 and nucleoprotein [26], nucleic acids [27–29], matrix protein [30], and serum amyloid A biomarker [31]. Biosensing technologies are constantly improved; the challenge is to create portable devices that overcome drawbacks related to long incubation times, unsatisfactory limits of detection, low stability, and short shelf life of the biosensor.

**Figure 1:**
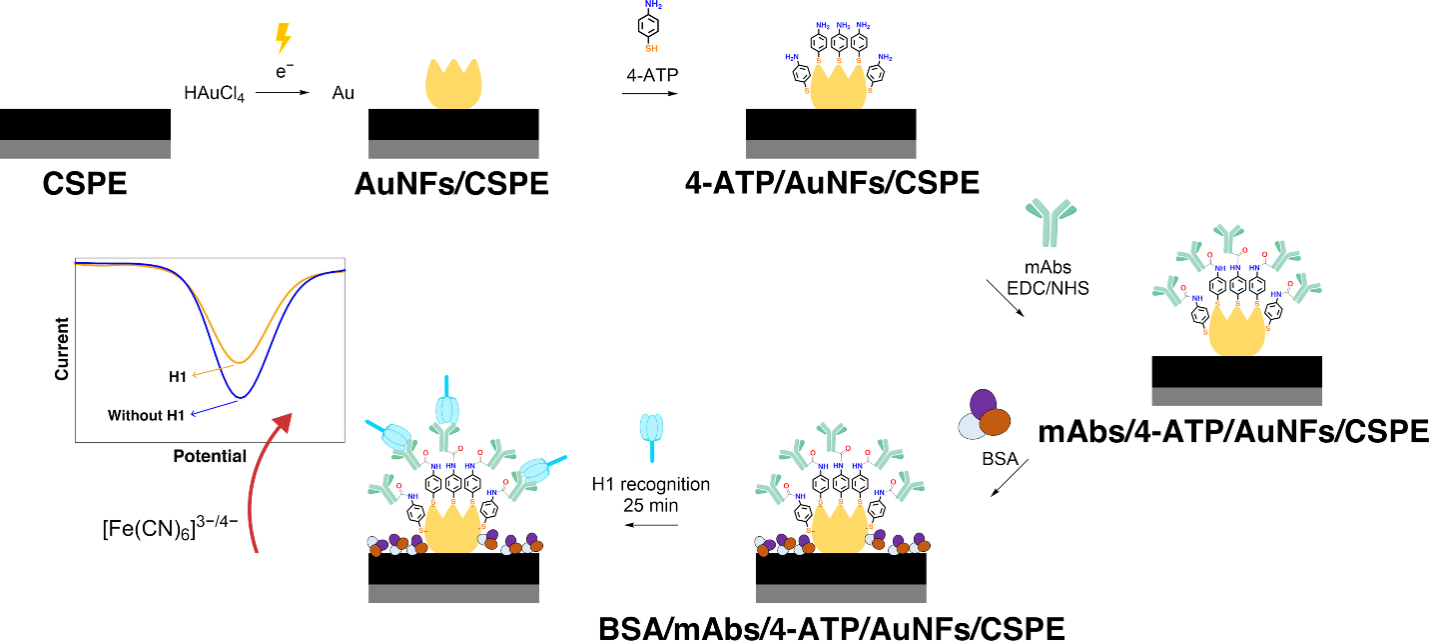
Preparation of the biosensing system and the effect on electrochemical current upon H1 protein recognition.

To address the current challenges, in this study, we built an electrochemical biosensor to detect and quantify H1 of influenza A H1N1 virus at clinically relevant concentrations with high accuracy and sensitivity in a complex matrix such as artificial saliva (Figure 1). The biosensing system can also be coupled to a microfluidics system without significant decrease in the electrochemical response. The transducer system of our biosensor is based on low-cost carbon screen printed electrodes (CSPEs) modified with functionalized gold nanoflowers (AuNFs). The complex morphology and surface functionalization of the nanoparticles with 4-aminothiophenol (4-ATP) significantly increased the surface area and the electron charge transfer at the surface of the electrode. To the best of our knowledge, this is the first time that the charge transfer enhancement with 4-ATP, a small organic molecule with delocalized π-electron system, has been employed to improve the sensitivity of electrochemical biosensing of proteins. This approach to amplify the electrochemical signal for biosensing of H1 provides a platform for the early detection of influenza A H1N1 virus.

## Results and Discussion

In this study, an electrochemical biosensor exhibiting enhanced electron charge transfer properties was constructed in order to detect the presence of the well-known biomarker H1 protein of influenza A H1N1 virus. This biosensor employs a differential pulse voltammetry technique to quantify H1 protein. The developed biosensor combines commercial electrodes with functionalized nanostructures and monoclonal antibodies to recognize H1 protein at clinically relevant concentrations.

### Scanning electron microscopy

**Figure 2:**
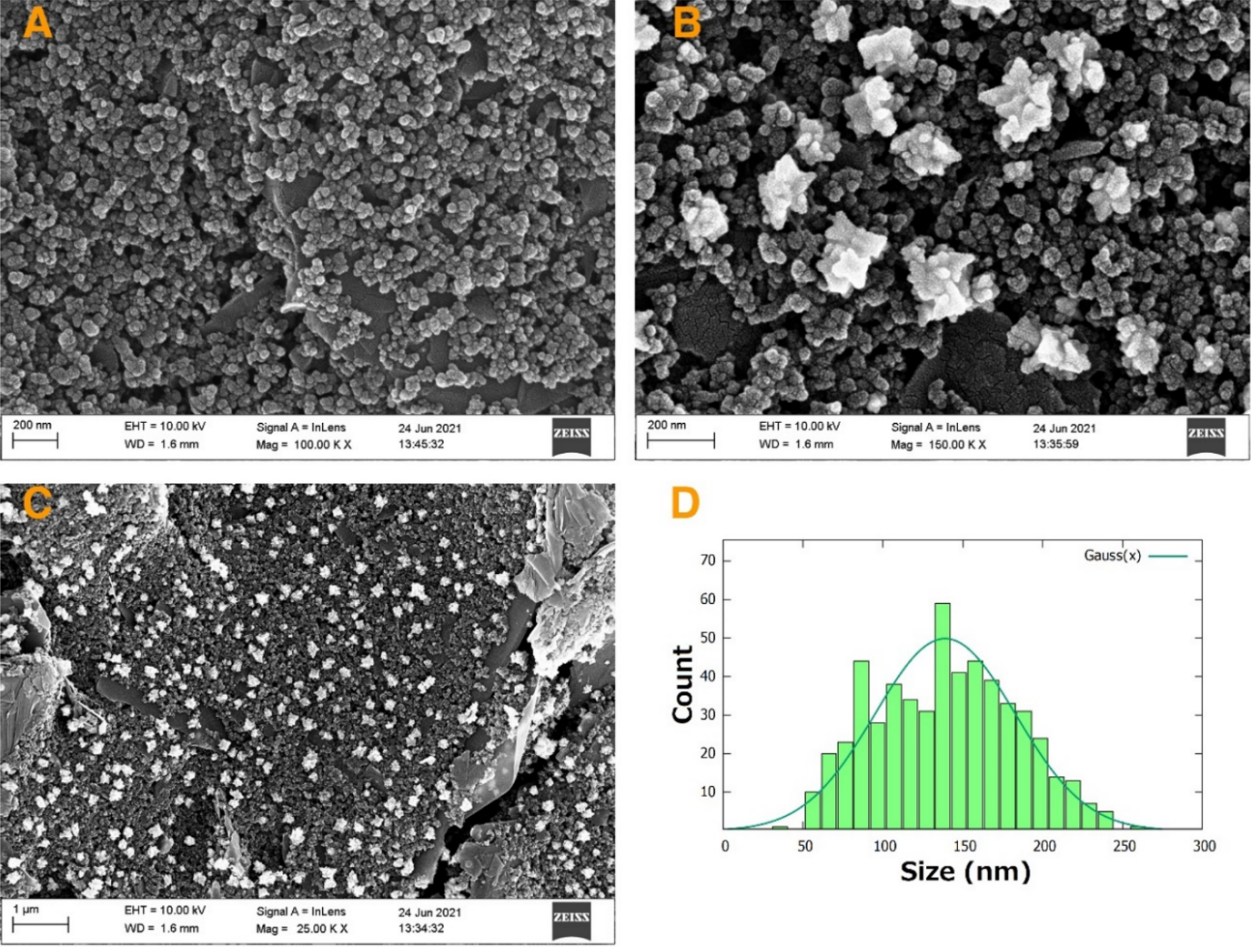
SEM images of (A) CSPE and (B, C) AuNFs/CSPE; (D) size distribution of the gold nanoflowers on AuNFs/CSPE.

Scanning electron microscopy (SEM) analysis was explored to characterize the surface of the electrodes after electrodeposition of gold nanoparticles (Figure 2). Because of the high conductivity of gold, a difference in contrast is observed when comparing the surface of the commercial CSPE (Figure 2A) to that of the AuNFs/CSPE (Figure 2B). The gold nanoparticles are evenly distributed across the surface of the electrode (Figure 2C). The deposited nanoparticles show a flower-like morphology with an average size of 139 nm and a standard deviation of 44 nm, which suggests size polydispersity (Figure 2D). The flower-like morphology of the nanostructures provides small Au domains across the electrode surface. The shape of these domains confers them with larger surface area than other types of nanostructures with plain geometric forms.

### Electrochemical characterization

**Figure 3:**
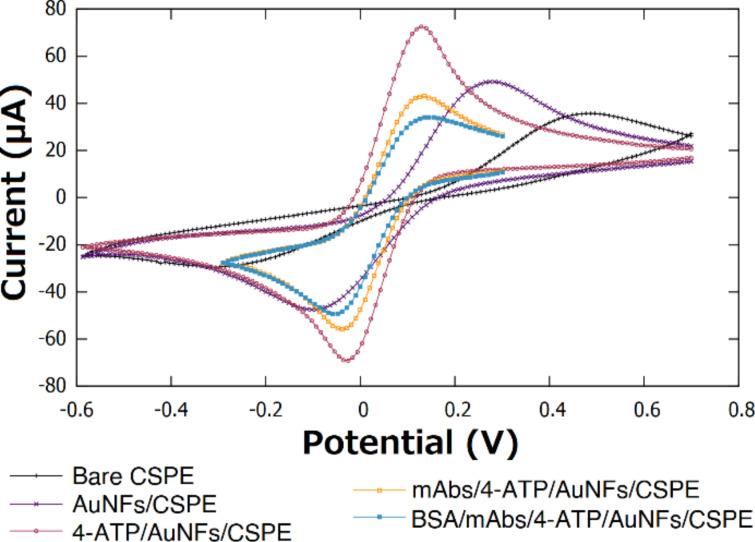
CV characterization at different steps of the electrode modification, measurements performed in 0.1 M KCl containing 5 mM [Fe(CN)_6_]^3−/4−^. The scan rate was 100 mV/s.

Although CSPEs have advantageous features such as low cost and wide availability, they tend to possess a characteristic high electrical resistance due to the use of inks containing organic molecules and polymeric binders during the fabrication process [32]. This could be seen experimentally (black curve of Figure 3), where the cyclic voltammetry (CV) analysis shows a large peak-to-peak separation of 0.72 V for the [Fe(CN)_6_]^3−/4−^ redox pair. This differs significantly from the theoretical 0.057 V peak-to-peak separation in reversible redox processes that involve one electron [33]. Typically, the sensing capabilities of electrochemical systems can be limited by the effective electroactive area of the electrode [34]. One approach to increase this parameter is the modification of electrodes with nanostructures that possess high surface area [35]. The strategy based on electrodeposition of gold nanoflowers increased the current response of the electrode because of the larger electroactive surface area compared to a CSPE (Figure 3). The CV analysis of the AuNFs/CSPE showed a peak-to-peak separation of 0.37 V. This value is smaller than in the commercial CSPE, which implies that electron transfer at the electrode surface was enhanced, increasing the redox reversibility for the [Fe(CN)_6_]^3−/4−^ pair. CSPEs are reported to possess a rough surface at the nanoscale [34]. The electrodeposition technique employed takes advantage of this to control the nucleation process, forming stable AuNFs that remain at the surface of the electrode upon contact with water and ethanol as no change in the CV was observed after contact with these solvents (Supporting Information File 1, Figure S1). This suggests strong mechanical adhesion of the AuNFs to the CSPE surface.

The largest change in the CV was observed after the functionalization of the AuNFs with 4-ATP through stable Au–S bonds. In this case, the peak-to-peak separation for the 4-ATP/AuNFs/CSPE was 0.16 V, showing that the redox reversibility for the [Fe(CN)_6_]^3−/4−^ pair and the current response of the electrode increased. This effect is known as tunneling charge transfer enhancement and significantly improved the sensitivity of the biosensor. It can be attributed to electron transfer through bonds due to the small length (0.59 nm) and the delocalized π-electron system of the 4-ATP linker molecule. Interestingly, the existence of this effect in a material appears to depend on the size of the superficial nanostructures. A similar effect has been reported for 4-ATP-functionalized multilayered nanostructures of Ag, Au, and Pt with a size range between 48 and 130 nm [36–37] as well as for 4-ATP-functionalized nanohybrids of MoSe_2_−CsPbBr_3_ with a size range between 60 and 80 nm [38]. This effect has been relevant to enhance the Raman scattering vibrational modes in surface-enhanced Raman spectroscopy measurements. It has also been noticed that the shape of the nanostructure can be used to tune the magnitude of the charge transfer enhancement by a factor of eight according to studies on spheres, tetrapods, cubes, and dogbone nanoparticles [36]. In contrast, no enhancement effect, or even slower charge transfer kinetics have been observed for 4-ATP-functionalized gold nanoparticles bearing sizes between 5 and 25 nm [39–43]. Therefore, the functionalization of the AuNFs with the linker 4-ATP represents one of the outstanding characteristics of our biosensing system. To the best of our knowledge, this is the first time that this enhancement effect has been explored to improve the sensitivity of electrochemical biosensing of proteins.

Covalent oriented immobilization of monoclonal antibodies (mAbs) was achieved through amide bond formation between terminal carboxylate moieties of mAbs and surface amine groups of the 4-ATP/AuNFs/CSPE. Using this strategy, the fragment crystallizable region of the mAbs is the section bound to the surface. This leads to proper antibody orientation such that the entire antigen binding site is available for adequate biorecognition [44]. No significant peak-to-peak separation change was observed in the CV analysis after immobilization of mAbs and blocking with bovine serum albumin (BSA) (Figure 3). This suggests that the final sensing platform preserved the favorable electrochemical properties achieved using AuNFs functionalized with 4-ATP.

**Figure 4:**
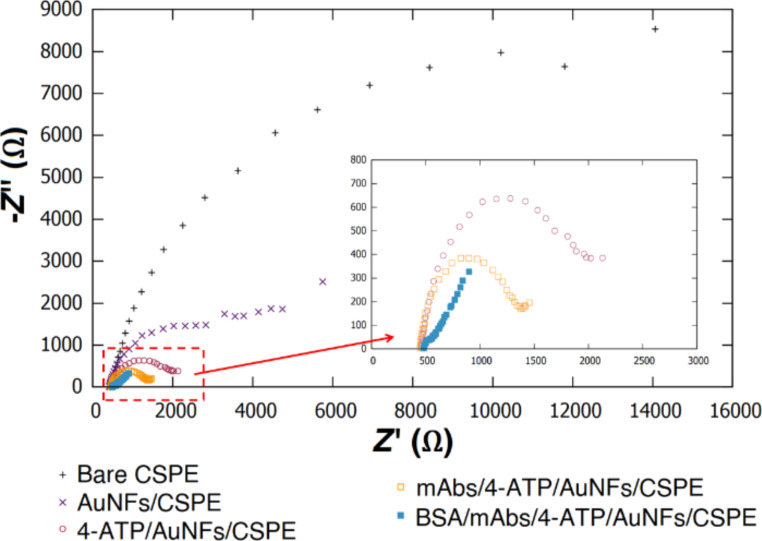
Electrical impedance spectroscopy measurements at different steps of electrode modification, recorded in 0.1 M KCl containing 5 mM [Fe(CN)_6_]^3−/4−^.

Electrochemical impedance spectroscopy (EIS) was performed to study the charge transfer processes at the surface of the modified electrode (Figure 4). In general terms, the elements of an electrochemical biosensor are analogous to the elements of an electric circuit [45]. A Randles equivalent circuit model was found to fit the experimental data obtained from the EIS analysis. This circuit contains a resistor (*R*_s_) to represent the ohmic resistance of the phosphate-buffered saline (PBS) electrolyte solution. The circuit is connected in series to the parallel combination of a capacitor (*C*_dl_) representing the double layer capacitance of the electrode–solution interphase and a resistor (*R*_ct_) accounting for the faradaic charge transfer resistance. A modulation of the *R*_ct_ magnitude was observed after each modification step on the working electrode of the biosensor (Supporting Information File 1, Figures S2–S6). Finally, the circuit is connected in series to a Warburg element (*W*_z_) representing the diffusion of the [Fe(CN)_6_]^3−/4−^ employed as redox probe in this study.

The magnitude of the *R*_ct_ of the electrodes at different modification stages was calculated by fitting the experimental Nyquist plots to the Randles equivalent circuit model. The commercial CSPE showed a high *R*_ct_ of 12.90 kΩ (Supporting Information File 1, Figure S2). Electrodeposition of AuNFs improved the electrochemical properties of the electrode by decreasing *R*_ct_ to 2.35 kΩ (Supporting Information File 1, Figure S3). The *R*_ct_ of the electrodes decreased to 126 Ω after functionalization with 4-ATP (Supporting Information File 1, Figure S4). These results indicated charge transfer enhancement at the surface of the 4-ATP/AuNFs/CSPE. In this system, *R*_ct_ was decreased by 100 times, a desired feature to improve the sensitivity of a biosensor. Immobilization of mAbs and blocking with BSA increased the *R*_ct_ to 825 Ω (Supporting Information File 1, Figure S5) and 1278 Ω (Supporting Information File 1, Figure S6), respectively.

**Figure 5:**
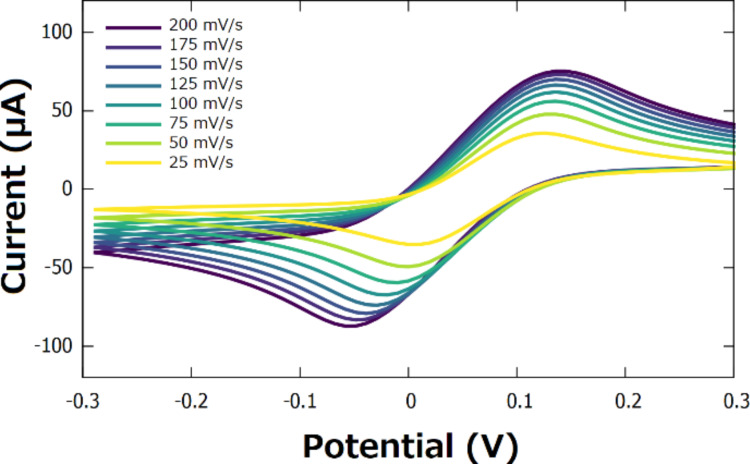
Cyclic voltammogram of the 4-ATP/AuNFs/CSPE at different scan rates, recorded in 0.1 M KCl containing 5 mM [Fe(CN)_6_]^3−/4−^.

**Figure 6:**
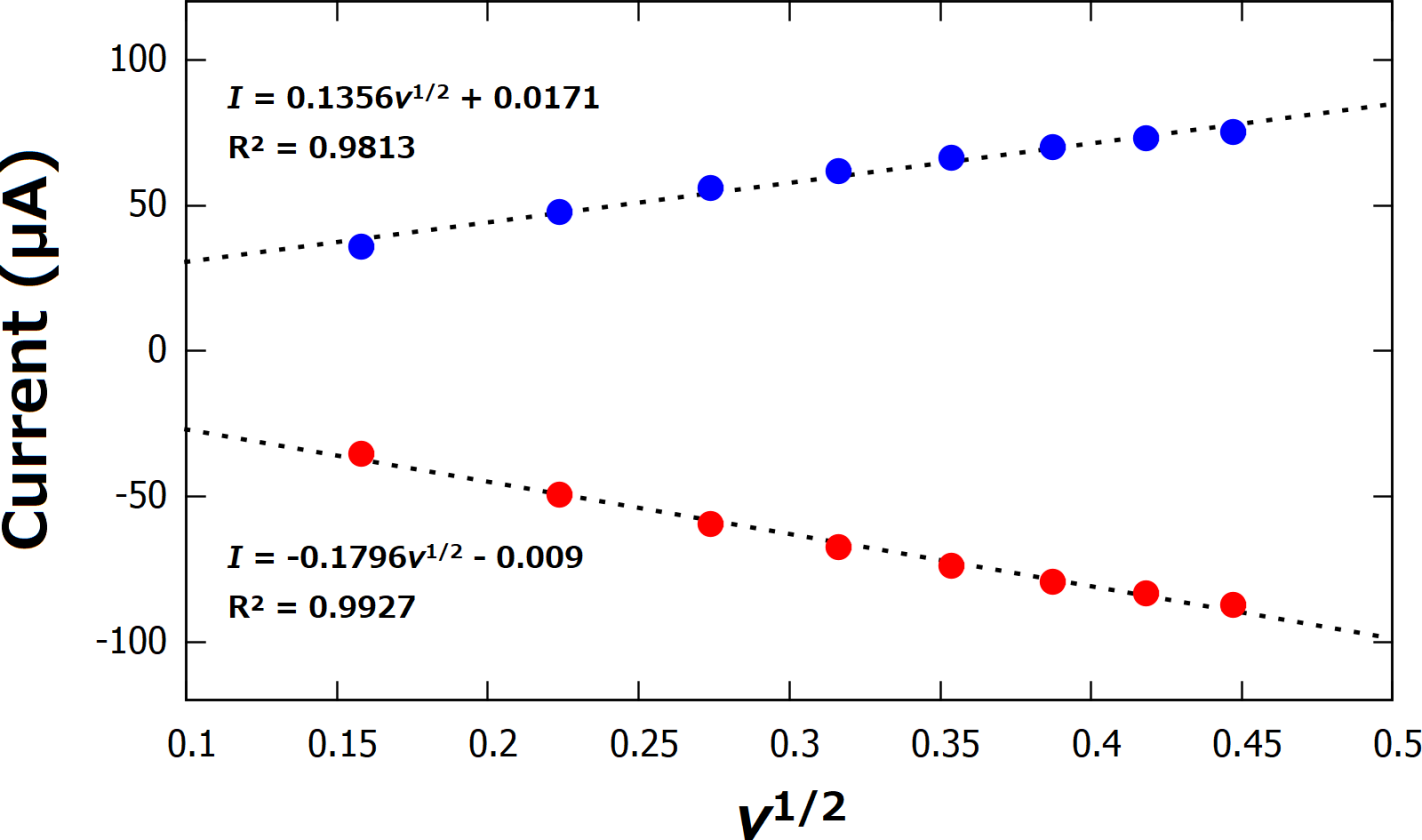
Linear models for the dependence of the current on the square root of the scan rate for the 4-ATP/AuNFs/CSPE.

The current response of the 4-ATP/AuNFs/CSPE in the presence of the electrochemical probe [Fe(CN)_6_]^3−/4−^ was studied using cyclic voltammetry at varying scan rates (Figure 5). Both the cathodic and anodic currents showed a linear correlation to the square root of the scan rate (Figure 6), suggesting that the reduction and oxidation of the complex [Fe(CN)_6_]^3−/4−^ is a diffusion-controlled process.

The performance of mouse monoclonal influenza A H1N1 hemagglutinin antibodies was tested using sandwich ELISA. They were selected as biorecognition element in our electrochemical biosensor after confirming the high specificity for hemagglutinin. In the ELISA, the limit of detection (LOD) of the viral H1 protein was determined to be 0.1 ng/mL.

### Biosensor response to standard solutions of H1 protein

**Figure 7:**
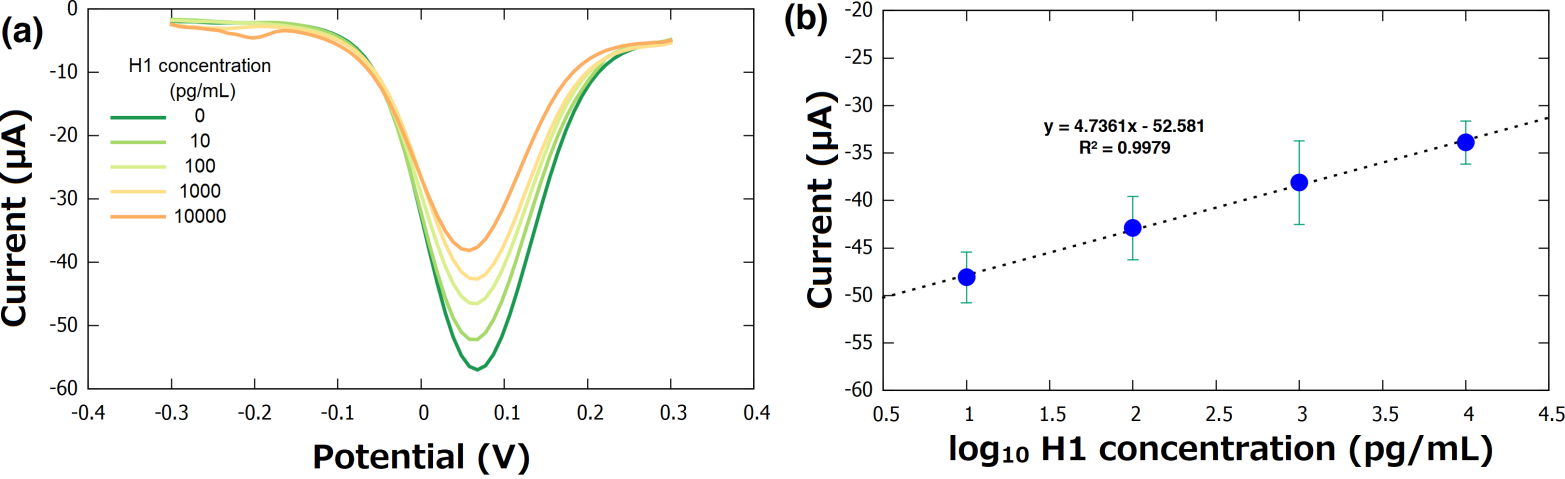
(a) Differential pulse voltammetry of the BSA/mAbs/4-ATP/AuNFs/CSPE, recorded in 0.1 M KCl containing 5 mM [Fe(CN)_6_]^3−/4−^ after 25 min exposure to artificial saliva standard solutions of H1 protein. (b) Calibration curve determined as the dependence of the change in current on the logarithm of the H1 protein concentration.

The biosensor was characterized using known concentrations of H1 protein dispersed in artificial saliva. Clinically relevant concentrations from 10 to 10,000 pg/mL were measured in a differential pulse voltammetry (DPV) experiment. Artificial saliva was used as a negative control to validate detection accuracy. The DPV technique was used for detection and quantification of antigen load because a good signal-to-noise ratio response was observed, making this detection a rapid and accurate process. Under the optimal parameters (pulse amplitude = 86 mV, potential increment = 4 mV, and scan rate = 100 mV/s), the negative control and four different concentrations (10, 100, 1,000 and 10,000 pg/mL) of viral surface protein H1 were measured with an incubation time of 25 min at RT. Experimentally, a significant difference between the generated current of the blank and the solutions containing H1 was observed (Figure 7a). Furthermore, a linear correlation was established between known sample concentration and current change in order to estimate H1 concentration in unknown samples. This correlation has a high *R*-square value of 0.9979 (Figure 7b).

### Microfluidics system

**Figure 8:**
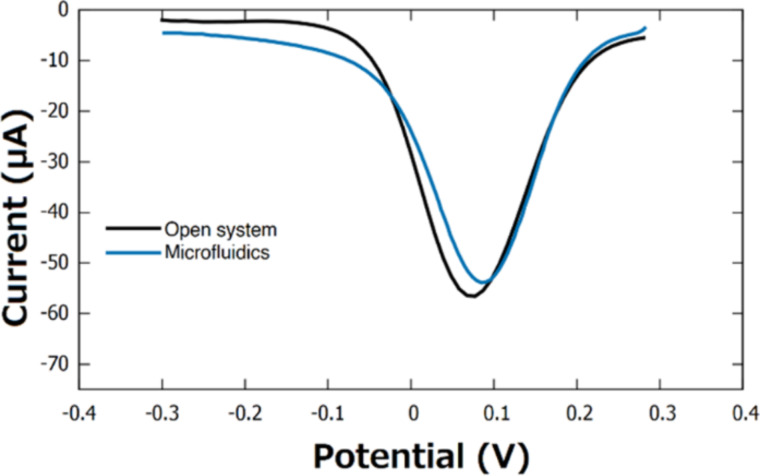
DPV measurement of the BSA/mAbs/4-ATP/AuNFs/CSPE recorded in 0.1 M KCl containing 5 mM [Fe(CN)_6_]^3−/4−^.

The final BSA/mAbs/4-ATP/AuNFs/CSPE was assembled with a microfluidics system. Upon exposition to H1 protein and measurement employing the standard DPV experiment (*n* = 3), a decrease in current response of 3.1% was observed (Figure 8) in comparison to a measurement without the microfluidics system. This decrease was probably due to a slight reduction in the surface area of the working electrode once the biosensor was assembled, sealed, and attached to the microfluidics system. The compatibility of biosensing devices with microfluidics systems is desired as this combination has the potential to miniaturize and shorten the acquisition time required to process a large number of biological samples, for example, in clinical measurements [46]. Furthermore, compatibility with microfluidic devices is important for the development of portable biosensing devices with automated functions and of simple use.

Different antigens of the influenza A H1N1 virus have been targeted for detection over the years. In order to make a substantial comparison, reported systems that targeted the H1 antigen are compared to our system in Table 1. The biosensing system developed in this work shows an acceptable LOD and can be included among biosensors for rapid detection (within minutes) of the H1 protein. In addition, the biosensing system of this work is also compared to other biosensors that employ the DPV technique to target either the hemagglutinin protein or the entire influenza A H1N1 virus. It is observed that the DPV technique offers acceptable LOD and rapid detection times, which are desirable features in biosensing systems.

**Table 1:** Comparison of the designed electrochemical biosensor to other systems from the literature that target influenza A H1N1 virus.

Biosensor detection technique	Analyte	Time to detection	LOD	Reference

colorimetry	hemagglutinin	12 h	11 pg/mL	[13]
DPV	hemagglutinin	1 min	9 pM (540 pg/mL)^a^	[14]
field-effect transistor	hemagglutinin	10 min	0.03 pg/mL	[18]
LSPR^b^	hemagglutinin	—	1 pM (60 pg/mL)^a^	[20]
ELISA	hemagglutinin	4 h	—	[47]
square wave voltammetry	whole virus	30 min	1.52 PFU/mL	[48]
DPV	hemagglutinin	30 min	3.7 PFU/mL	[49]
DPV	whole virus	30 min	0.76 pg/mL	[50]
DPV	whole virus	12 h	0.25 pg/mL	[51]
DPV	whole virus	10 min	100 ng/mL	[52]
DPV	hemagglutinin	25 min	19 pg/mL	this work

^a^The molecular weight of H1 is 60 kDa [53]; ^b^LSPR = local surface plasmon resonance.

The biosensor developed in this study can be manufactured with antibodies targeting pandemic influenza strains such as the latest H1N1 that emerged in 2009, also known as the Swine Flu. Since then, H1N1 has been circulating in the community together with other seasonal influenza strains, and surveillance of this virus is required [54]. Furthermore, because of the potential advantage of changing specific monoclonal antibodies in the system, it can easily be adjusted for any and multiple seasonal influenza strains, similar to the case of annual influenza vaccines, which are updated every year to match the currently circulating viruses [55]. Additionally, this characteristic could allow our biosensing platform to be used in mass testing in a short period of time, which is crucial for controlling the spread of a virus among the population. This biosensing platform has the potential to be adapted to target other respiratory viruses such as SARS-CoV-2, a virus for which it has been shown that it can be detected in saliva samples [55].

Our biosensing system for the detection of hemagglutinin protein of influenza A H1N1 virus is currently limited to solutions of the H1 protein in artificial saliva. Future validation tests with clinical samples containing intact virus particles will reveal the potential of this biosensor to be applied for the rapid detection of the influenza A virus. We believe that the use of clinical samples will enable increased sensitivity due to the size and weight of the intact virus (as compared to the H1 protein itself), as well as due to the presence of large number of H1 molecules on the surface of the viral particles.

## Conclusion

In this study, a label-free biosensing tool for the detection of hemagglutinin protein of the H1N1 influenza A virus was developed. We have modified low-cost carbon screen-printed electrodes with gold nanoflowers via electrodeposition, functionalized the gold nanoflowers with 4-aminothiophenol, immobilized monoclonal antibodies that specifically target H1 protein, and used BSA to prevent non-specific binding. Differential pulse voltammetry was used in the electrochemical detection of H1 in artificial saliva revealing that the biosensor performs with good reproducibility and sensitivity in the clinically relevant concentration range. The LOD for hemagglutinin is 19 pg/mL, and a good correlation between hemagglutinin concentration and peak current was observed in the concentration range from 10 to 10 000 pg/mL. The experimental EIS data suggest that the electron transfer on the electrode was enhanced by a factor of 100 due to the increase in surface area and to a tunneling charge transfer effect. This improvement is attributed to the synergistic effect of the electrodeposited gold nanoflowers and the functionalization with 4-aminothiophenol. Furthermore, the developed biosensor can be attached to a 3D-printed microfluidic system to be used as a point-of-care device without any significant deleterious effect on the electrochemical performance of the biosensor.

## Experimental

### Reagents and Materials

Carbon screen-printed electrodes (CSPEs) were obtained from Zimmer & Peacock (Norway). Hemagglutinin protein of influenza A H1N1 virus (H1) and mouse monoclonal antibodies (mAbs) were purchased from Sinobiological (Germany). Secondary goat anti-rabbit IgG antibodies Alexa Flour 568 were obtained from ThermoFisher (USA). Artificial saliva was obtained from LCTech GmbH (Germany). Syringeless filters of regenerated cellulose membrane (0.45 μm) were purchased from Cytiva (Sweden). Chloroauric acid (HAuCl_4_), hydrochloric acid, sulfuric acid, 4-aminothiophenol (4-ATP), ethanol, potassium chloride, potassium hexacyanoferrate(II) trihydrate, potassium hexacyanoferrate(III), *N*-(3-dimethylaminopropyl)-*N*′-ethylcarbodiimide hydrochloride (EDC), *N*-hydroxy succinimide (NHS), phosphate-buffered saline pH 7.4 (PBS), and bovine serum albumin (BSA) were all purchased from Sigma-Aldrich (Germany) and were used without further purification.

### Electrodeposition of gold nanoflowers

The electrodeposition of AuNFs was carried out to increase the surface area of the electrodes. The AuNFs were synthesized following a method from the literature [56] with some modifications. Briefly, 50 μL of a 2 mM HAuCl_4_ solution containing 6 mM HCl and 0.5 M sulfuric acid was added on top of the CSPE, and a potential of −0.25 V (vs Ag/AgCl) was applied for 60 s. The electrode was then rinsed with 25 mL deionized water, dried under a flow of N_2_, and stored at room temperature (RT) in dark.

### Functionalization with 4-ATP

The surface of the electrodes was functionalized to introduce an amine group, which was used to covalently bind the mAbs as biorecognition element. The molecule 4-ATP possesses a thiol group capable of self-assembling on the surface of the AuNFs. A method from the literature [57] with some modifications was employed. Briefly, the working electrode was covered with 10 μL of 10 mM 4-ATP solution in ethanol and incubated at 22 °C for 15 min. Unreacted 4-ATP was removed by two consecutive washings with 1 mL of ethanol and 1 mL of PBS, respectively. The 4-ATP/AuNFs/CSPE electrode was then dried under a flow of N_2_ gas and stored at 4 °C until use.

### Immobilization of mAbs

The mAbs are essential in our biosensor and function as biorecognition element toward H1 protein. The mAbs were immobilized as described previously [58] with some modifications. Briefly, a reaction mixture of 300 μL was prepared in a 1.5 mL Eppendorf vial by adding 100 μL of 38 μg/mL mABs solution in PBS, 100 μL of 10 mM EDC aqueous solution, and 100 μL of 10 mM NHS aqueous solution. The pH of the mixture was 6.5 as determined by litmus paper. Then, 10 μL of the mixture was added on top of the 4-ATP/AuNFs/CSPE as working electrode and incubated at 4 °C overnight. Afterwards, the mAb/4-ATP/AuNFs/CSPE was rinsed with 1 mL of PBS to remove unreacted species and dried under a flow of N_2_ gas. The surface of the electrode was blocked by adding 10 μL of 0.5% BSA solution in PBS and incubated at 4 °C for 2 h. Thereafter, the BSA/mAb/4-ATP/AuNFs/CSPE was rinsed with 1 mL of PBS, dried under a flow of N_2_, and stored at 4 °C.

### Quantification of hemagglutinin

A serial dilution of H1 from 10 to 10,000 pg/mL spiked in artificial saliva were prepared from a 100 μg/mL stock solution in PBS. A solution of artificial saliva with no hemagglutinin was used as negative control. After filtrating the solutions through a 0.45 μm membrane filter to remove any suspended particles, 50 μL were deposited on the functionalized electrode and incubated at RT for 25 min. The electrode was then rinsed with 1 mL of PBS and dried under a flow of N_2_ gas. Subsequently, 50 μL of a 5 mM [Fe(CN)_6_]^3−/4−^ in 0.1 M KCl solution were used to cover the electrode, and a DPV experiment was performed to characterize the surface of the electrode.

The obtained voltammograms were used to generate a calibration curve, in which the change in current is proportional to the logarithm of the concentration of H1 in the solution. The limit of detection (LOD) was calculated following a conventional criterion where LOD is equal to the mean of the signal of a blank solution plus three standard deviations [39]. The mean of the signal was obtained from a series of DPV experiments after exposing the biosensor to blank solutions of artificial saliva without H1 protein.

### Electrochemical measurements

The commercial CSPE employed in this study consists of a three-electrode cell array with a carbon working electrode, a Ag/AgCl reference electrode, and a carbon counter electrode. Potentiostat EmStat Pico with PSTrace 5.8 software was employed to carry out CV, EIS, and DPV experiments. All the experiments were performed in the presence of 5 mM [Fe(CN)_6_]^3−/4−^ in 0.1 M KCl solution. EIS measurements were performed applying a 6 mV potential at different frequencies from 5 mHz to 50 kHz. DPV measurements were performed with equilibration of 5 s, scanning potential from 0.3 to −0.3 V (vs Ag/AgCl), *E*_step_ = 0.01 V, *E*_pulse_ = 0.086 V, and *t*_pulse_ = 4 s at a scan rate of 100 mV/s.

### SEM analysis of AuNFs

Prior to SEM imaging, the electrode samples were sputtered with a layer of gold/palladium for 40 s at 2 kV (Emitech SC7640, Quorum technologies). Representative micrographs of the AuNFs were taken using a secondary in-lens detector at a working distance of 1.6 mm and an acceleration voltage of 10 keV (Ziess LEO 1530, AB Carl Zeiss). The size distribution of the AuNFs was calculated by measuring the AuNFs in the micrograph presented in Figure 2c, a total of 540 AuNFs were identified and measured with the open-source ImageJ software [59].

### Microfluidics system

**Figure 9:**
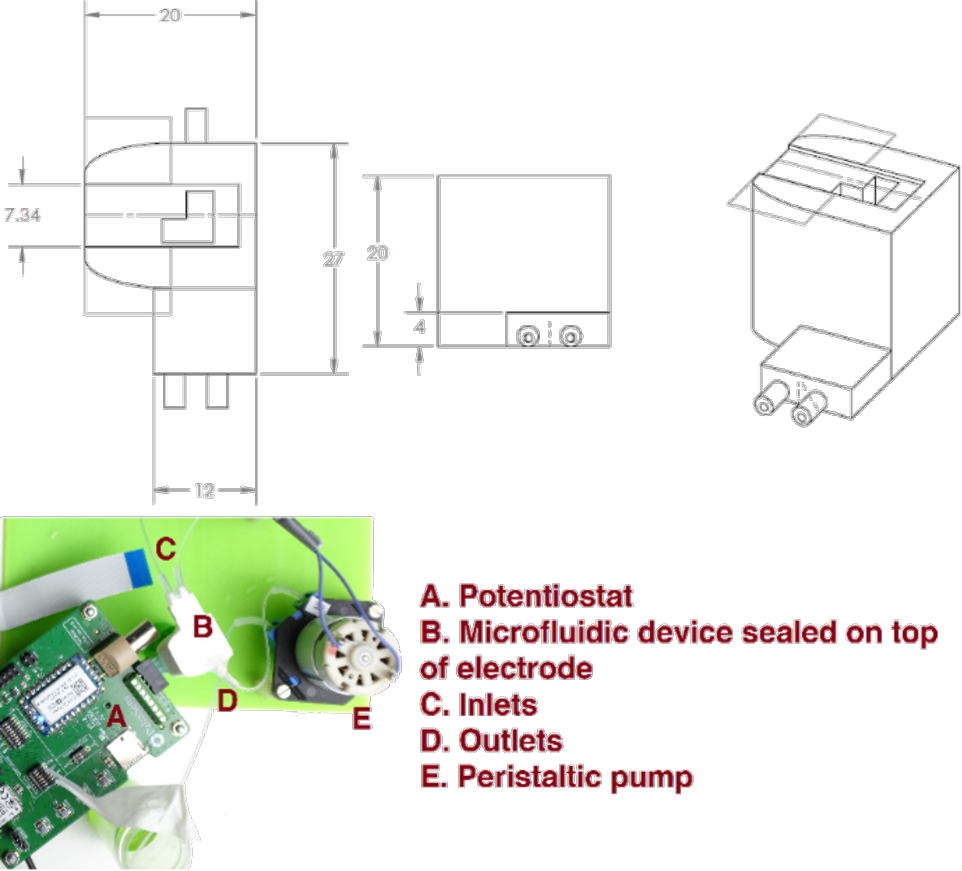
Microfluidics design for point-of-care applications, the dimensions are in millimeters, and a photograph of the entire device is shown.

A microfluidics device (Figure 9) was designed to use the biosensor in point-of-care applications. This system was coupled to a peristaltic pump, and it allowed us to automate the addition of the sample and the reagents to the electrodes during the quantification of hemagglutinin. The microfluidic device, which was printed using a 3D printer (Ultimaker 2+, Ultimaker), contained a slot through which the electrode can be placed and sealed in place using silicon glue (Elastosil A07, Wacker). The device also consisted of two inlets and one outlet through which reagents can be made to pass through the device. The inlets and outlets were connected to reagent sources using silicon tubing (inner diameter = 1 mm, VWR).

## Supporting Information

File 1 Additional experimental details, electrode stability in different solvents, and circuit fitting for the EIS data.

## Data Availability

All data that supports the findings of this study is available in the published article and/or the supporting information of this article.
