# Supplementary material for: Functionalized gold nanoflowers on carbon screen-printed electrodes: an electrochemical platform for biosensing hemagglutinin protein of influenza A H1N1 virus
Source: Beilstein J Nanotechnol. 2025 Apr 16;16:540–50. doi: 10.3762/bjnano.16.42 (PMC12018907; doi:10.3762/bjnano.16.42)
Supplement: File 1 — Additional experimental details, electrode stability in different solvents, and circuit fitting for the EIS data. [file Beilstein_J_Nanotechnol-16-540-s001.pdf]

BEILSTEIN JOURNAL OF NANOTECHNOLOGY

Supporting Information

for

Functionalized gold nanoflowers on carbon screen-printed electrodes: an electrochemical platform for biosensing hemagglutinin protein of influenza A H1N1 virus

Carlos Enrique Torres-Méndez, Sharmilee Nandi, Klara Martinovic, Patrizia Kühne, Yifan Liu, Sam Taylor, Maria Lysandrou, Maria Ines Berrojo Romeyro Mascarenhas, Viktoria Langwallner, Javier Enrique Sebastián Alonso, Ivana Jovanovic, Maike Lüftner, Georgia-Vasiliki Gkountana, David Bern, Abdul-Raouf Atif, Ehsan Manouchehri Doulabi, Gemma Mestres and Masood Kamali-Moghaddam

Beilstein J. Nanotechnol. **2025,** *16,* 540–550. doi:10.3762/bjnano.16.42

Additional experimental details, electrode stability in different solvents, and circuit fitting for the EIS data

License and Terms: This is a supporting information file under the terms of the Creative Commons Attribution License (https://creativecommons.org/licenses/by/4.0). Please note that the reuse, redistribution and reproduction in particular requires that the author(s) and source are credited and that individual graphics may be subject to special legal provisions.
The license is subject to the *Beilstein Journal of Nanotechnology* terms and conditions: (https://www.beilstein-journals.org/bjnano/terms)

Validation of mAb specificity and sensitivity. Sandwich enzyme-linked immunosorbent assay (ELISA) was performed to characterize specificity and sensitivity of the monoclonal antibodies; a protocol from the company providing the monoclonal antibodies was followed. In short, in a polyvinylchloride microtiter, 100 µL of antibody solution (0.228 µg/mL) was added to each well of the microtiter. The plate was incubated overnight at 4 °C to allow for antibody binding to the bottom of the wells. The wells were washed thrice with PBS solution. The wells were filled with 200 µL of 3% BSA/PBS. The plate was incubated overnight in a humid atmosphere at room temperature. The wells were washed thrice with PBS solution. Subsequently, 100 µL of the H1 solution (1 µg/mL) in 3% BSA/PBS was added to each well. The plate was washed thrice with PBS. The solution containing secondary antibody was added (100 µL). The plate was incubated for 30 min at 37 °C in the dark. The plate was washed five times with PBS. The substrate was added, the plate was incubated, and the optical density at 568 nm was measured on an ELISA plate reader.

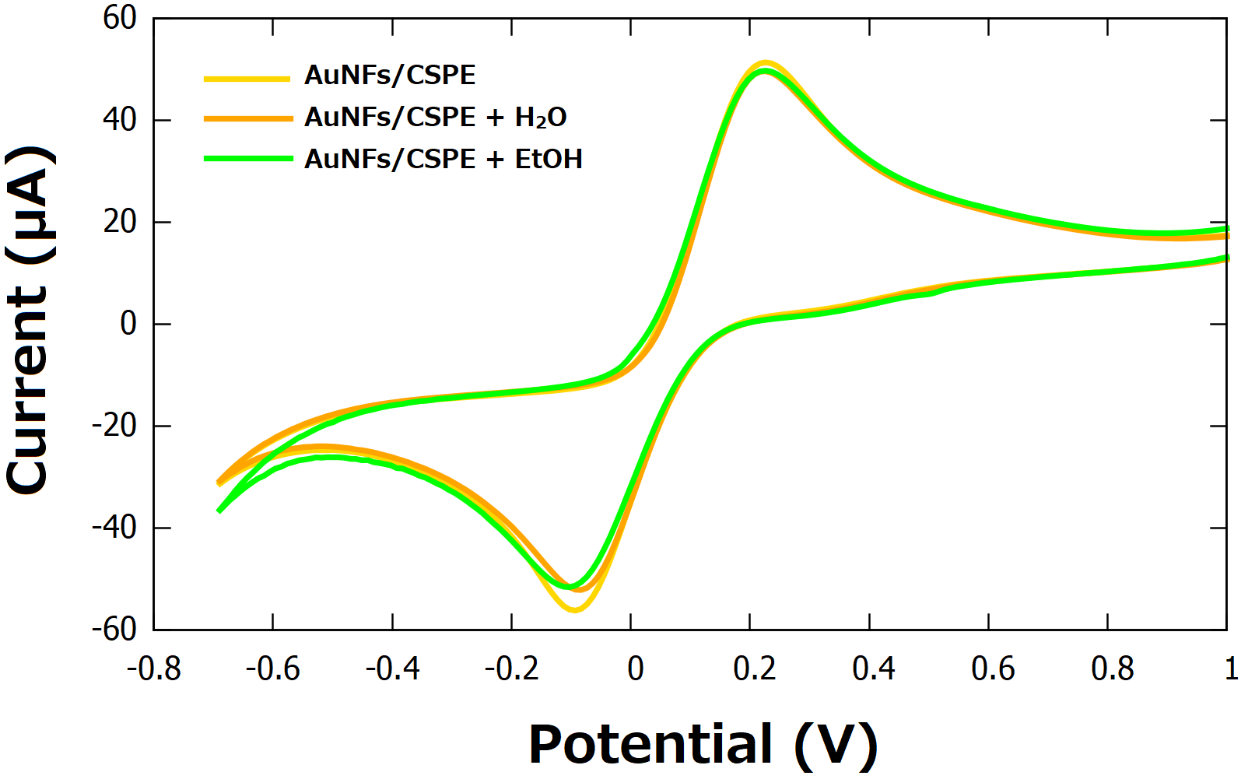

Figure S1: CV of AuNFs/CSPE upon addition of water and ethanol, recorded in 0.1 M KCl containing 5 mM $[Fe(CN)_6]^{3-/4-}$, with a scan rate of 100 mV/s.

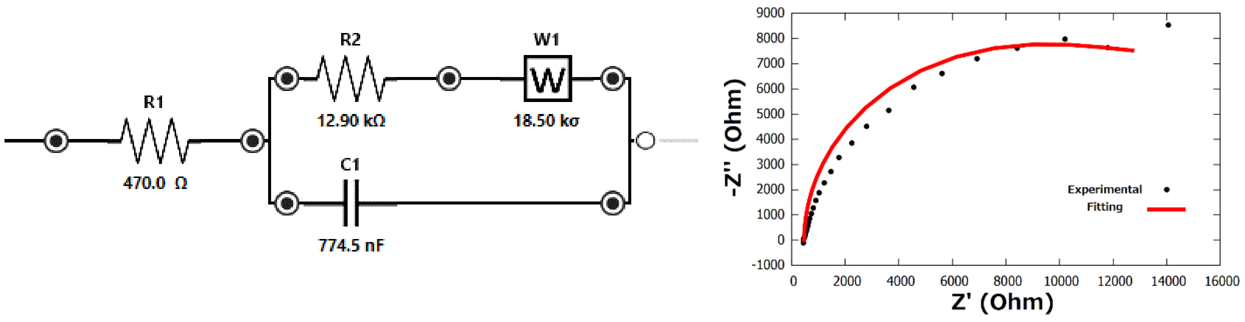

Figure S2: Equivalent circuit and Nyquist plot for the CSPE.

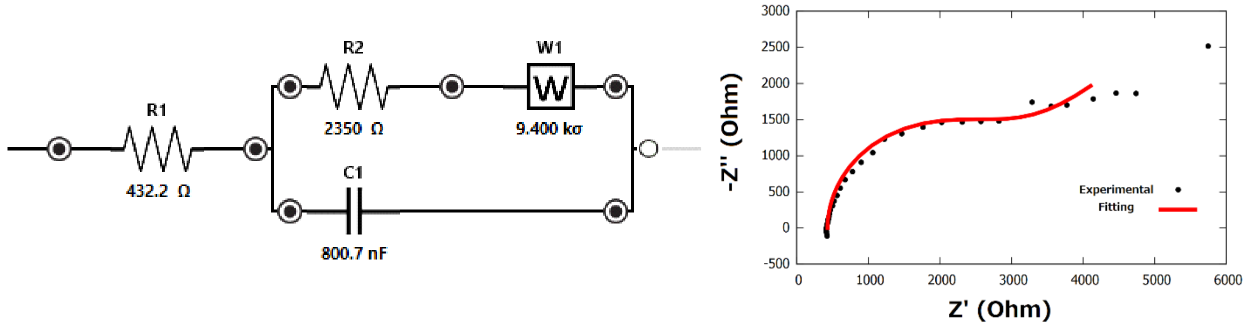

Figure S3: Equivalent circuit and Nyquist plot for the AuNFs/CSPE.

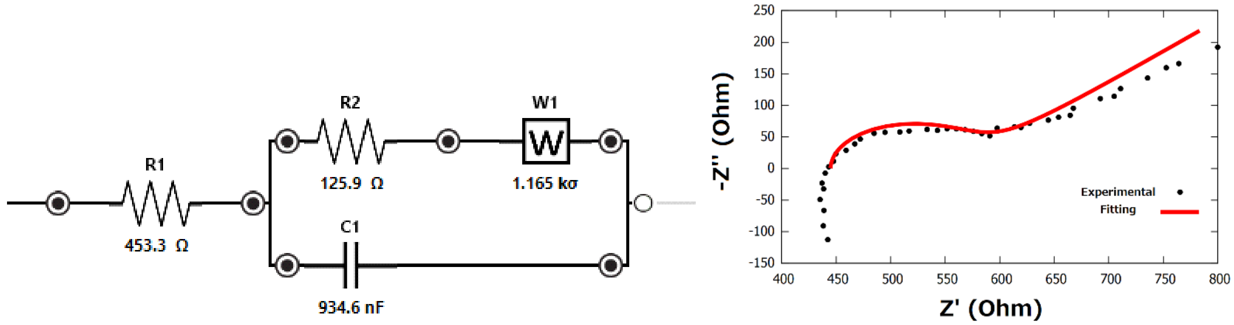

Figure S4: Equivalent circuit and Nyquist plot for the 4-ATP/AuNFs/CSPE.

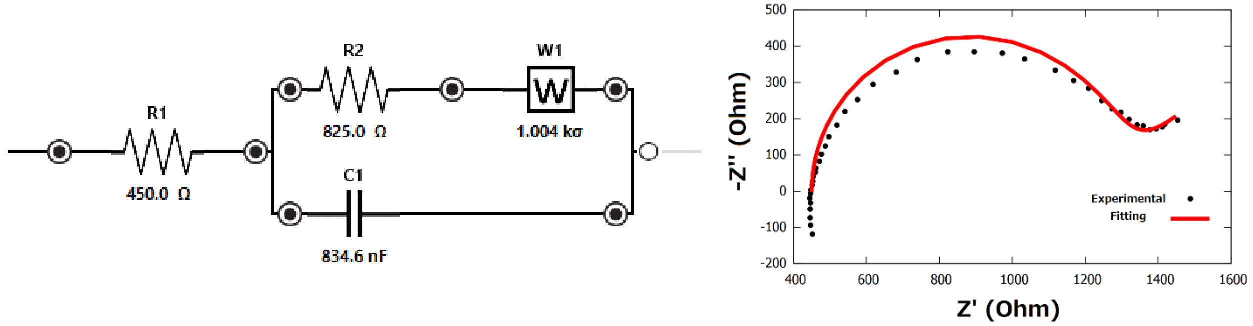

Figure S5: Equivalent circuit and Nyquist plot for the mAbs/4-ATP/AuNFs/CSPE.

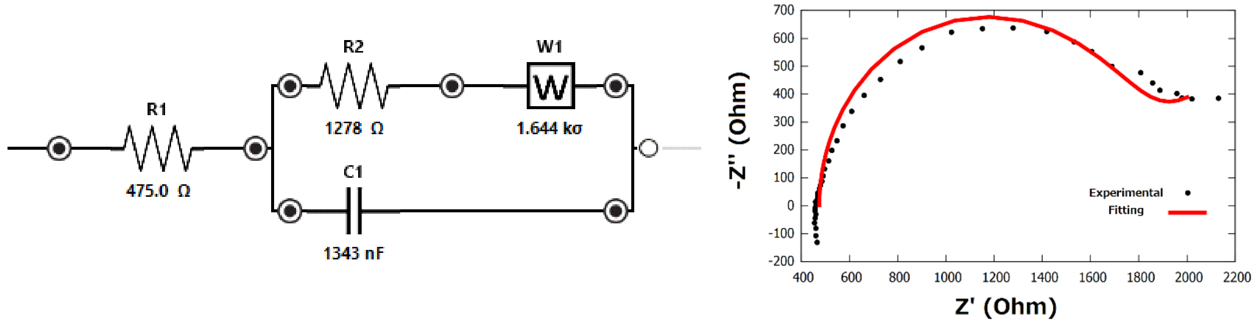

Figure S6: Equivalent circuit and Nyquist plot for the BSA/mAbs/4-ATP/AuNFs/CSPE.

Table S1: Mouse monoclonal influenza A H1N1 (Swine Flu 2009) hemagglutinin antibody specificity in ELISA provided by SinoBiological.

**Cross-reactivity**	**No cross-reactivity**
H1N1 (A/BrevigMission/1/1918) HA	H1N1 (A/Brisbane/59/2007) HA
H1N2 (A/swine/Guangxi/13/2006) HA	H1N1 (A/Solomon Islands/3/2006) HA
H1N3 (A/duck/NZL/160/1976) HA	H1N1 (A/Ohio/UR06-0091/2007) HA
	H1N1 (A/New Caledonia/20/1999) HA
	H1N1 (A/Puerto Rico/8/1934) HA
	H1N1 (A/WSN/1933) HA
	H3N2 (A/Brisbane/10/2007) HA
	H5N1 (A/Anhui/1/2005) HA
	H5N1 (A/Indonesia/5/2005) HA
	H5N1 (A/Vietnam/1194/2004) HA
	H5N1 (A/bar-headed goose/Qinghai/14/2008) HA
	H5N1 (A/turkey/Turkey/1/2005) HA
	Influenza B (B/Florida/4/2006) HA
